# Gene expression signatures for colorectal cancer microsatellite status and HNPCC

**DOI:** 10.1038/sj.bjc.6602621

**Published:** 2005-05-24

**Authors:** M Kruhøffer, J L Jensen, P Laiho, L Dyrskjøt, R Salovaara, D Arango, K Birkenkamp-Demtroder, F B Sørensen, L L Christensen, L Buhl, J-P Mecklin, H Järvinen, T Thykjaer, F P Wikman, F Bech-Knudsen, M Juhola, N N Nupponen, S Laurberg, C L Andersen, L A Aaltonen, T F Ørntoft

**Affiliations:** 1 Molecular Diagnostic Laboratory, Department of Clinical Biochemistry, Aarhus University Hospital, Brendstrupgaardsvej 100, DK-8200, Aarhus, Denmark; 2 Department of Statistics, Aarhus University, Aarhus, Denmark; 3 Department of Medical Genetics, Haartman Institute, University of Helsinki, Helsinki, Finland; 4 Institute of Pathology, Aarhus University Hospital, Brendstrupgaardsvej 100, DK-8200, Aarhus, Denmark; 5 Department of Pathology, Jyväskylä Central Hospital, Jyväskylä, Helsinki, Finland; 6 University Central Hospital, Haartman Institute, University of Helsinki, Helsinki, Finland; 7 AROS Applied Biotechnology ApS, Research Park Skejby. Aarhus. Denmark; 8 Department of Surgery, Aarhus University Hospital, Brendstrupgaardsvej 100, DK-8200, Aarhus, Denmark

**Keywords:** colon cancer, gene expression, microsatellite instability, HNPCC

## Abstract

The majority of microsatellite instable (MSI) colorectal cancers are sporadic, but a subset belongs to the syndrome hereditary nonpolyposis colorectal cancer (HNPCC). Microsatellite instability is caused by dysfunction of the mismatch repair (MMR) system that leads to a mutator phenotype, and MSI is correlated to prognosis and response to chemotherapy. Gene expression signatures as predictive markers are being developed for many cancers, and the identification of a signature for MMR deficiency would be of interest both clinically and biologically. To address this issue, we profiled the gene expression of 101 stage II and III colorectal cancers (34 MSI, 67 microsatellite stable (MSS)) using high-density oligonucleotide microarrays. From these data, we constructed a nine-gene signature capable of separating the mismatch repair proficient and deficient tumours. Subsequently, we demonstrated the robustness of the signature by transferring it to a real-time RT-PCR platform. Using this platform, the signature was validated on an independent test set consisting of 47 tumours (10 MSI, 37 MSS), of which 45 were correctly classified. In a second step, we constructed a signature capable of separating MMR-deficient tumours into sporadic MSI and HNPCC cases, and validated this by a mathematical cross-validation approach. The demonstration that this two-step classification approach can identify MSI as well as HNPCC cases merits further gene expression studies to identify prognostic signatures.

Colorectal cancer is a major public health problem as it accounts for about 13% of all cancers and is the second most common cause of cancer death in the western world (Greenlee *et al*, 2001∣Parkin, 2001). Tumour stage is the main determinant of outcome for these cancer patients as it is for most cancer patients. About 15% of colorectal cancers exhibit microsatellite instability (MSI) and these are reported to have a good prognosis relative to microsatellite stable (MSS) patients (Lothe *et al*, 1993∣Bubb *et al*, 1996).

Microsatellite instability was first identified in hereditary nonpolyposis colorectal cancer (HNPCC) families and found to be caused by mutations in the *MLH1* or *MSH2* genes, leading to failures in the mismatch repair (MMR) system. In sporadic MSI cases, the main cause of MMR failure was later found to be silencing of the MLH 1 promoter by methylation of CpG islands (Herman *et al*, 1998). The MMR-deficient tumours have characteristic features like being poorer differentiated, having more inflammation, and a more proximal location (Kane *et al*, 1997; Ropponen *et al*, 1997; Cunningham *et al*, 1998b). At many hospitals tumours are screened for MMR deficiency using microsatellites and/or immunohistochemistry and those that are MSI are evaluated for germline mutations in the *MMR* genes.

A new diagnostic approach is molecular classification of tumours based on DNA microarrays, that has shown the ability to produce gene expression signatures with predictive power for a variety of tumour types (reviewed by Dyrskjot, 2003). In a disease like bladder cancer, several classifiers have been published predicting upstaging, recurrence, and surrounding field disease (Dyrskjot, 2003).

A few reports have analysed global gene expression patterns in colorectal tumours with focus on microsatellite status. Mori *et al* (2003) found that MSI had a great impact on the global phenotype and Banerjea *et al* (2004) identified a gene expression cluster in MSI tumours that correlated with an activated immune response. The aim of the present study was to generate expression profiles from a broad spectrum of colorectal tumours in order to identify a robust gene signature that could separate between MSI and MSS. This could be the first step towards a stricter definition of molecular subgroups of colon cancer that may be necessary in the effort to construct clinically useful signatures for prognosis and response to chemotherapy. We built a maximum likelihood MSI classifier using 101 tumour expression profiles and evaluated it using a leave-one-out cross-validation scheme. The classifier was then validated using RT-PCR on an independent test set of 47 tumours. In the second step, we investigated the microsatellite unstable tumours separately, and identified two genes that separated sporadic MSI from inherited cases.

## MATERIALS AND METHODS

### Patients and biopsy specimens

The tumours included in this study were resected at 15 different clinics in Denmark and Finland. The study was approved by the local ethic committees of all clinics, and all patients gave informed consent prior to surgery. Colorectal cancer tissue from a total of 151 patients was collected and embedded in either Tissue Tek Oct-compound or a SDS/guadinium thiocyanate solution and frozen immediately after surgery. On occasions normal mucosa biopsies were also collected and 17 of these were included in the study. In a few instances biopsies were frozen directly without any prior embedding.

A sample set consisting of 101 stages II and III cancers (34 MSI and 67 MSS) and 17 normal mucosa samples were used for gene expression profiling. To enable the construction of a general MMR gene expression signature, caution was paid to avoid over-representation of a particular subtype of MSI tumour. Thus, MSI tumours of both sporadic and HNPCC origin were selected. The histology subtypes of the MSI tumours were selected to cover both ordinary and mucinous adenocarcinomas. Special attention was also paid to select cancers of the right and left colons as well as the rectum. Similarly, the normal samples were both from MSI and MSS patients (three MSI, four MSS, 10 not determined) and represented both the right and left colons. A brief summary of the sample set used for gene expression profiling can be found in Table 1.

An independent sample set consisting of 47 stage II cancers (10 MSI and 37 MSS) was used for real-time RT-PCR (Table 2), and served as independent test set.

The analysis of MSI origin (sporadic or HNPCC) was performed on a sample set consisting of 37 MSI cancers (34 from the gene expression profiling sample set plus an additional three new stage I and IV MSI cancers). All HNPCC cases included in this study carry MLH1 (*n*=16) or MSH2 (*n*=2) mutations identified by sequencing.

#### Microsatellite analysis

Tumour DNA was extracted from gross dissected cancer tissue. Control DNA was extracted from blood samples when available and normal epithelium, from the oral resection edge, otherwise. Microsatellite instability was determined using a pentaplex polymerase chain reaction with five quasimonomorphic mononucleotide repeats, as previously described (Suraweera *et al*, 2002). Tumours with low-frequency MSI have similar clinical features as MSS tumours and were considered as such in this study.

#### RNA purification

Total RNA was isolated using Trizol (Invitrogen) or GenElute Kits (Sigma) according to the manufacturers' instructions. RNA integrity was evaluated on a 2100 Bioanalyzer using the RNA 6000 Nano LabChip kit (Agilent). Only samples with intact RNA were used for gene expression analysis.

#### Gene expression analysis

Labelling of RNA, hybridisation and scanning was performed as described elsewhere (Dyrskjot *et al*, 2003). Biotin-labelled cRNA was prepared from 10 *μ*g of total RNA and hybridised to the Human Genome U133A GeneChip array (Affymetrix). This array contains 22 289 probesets representing approximately 15 000 genes. The readings from the quantitative scanning were analysed by the Affymetrix Software MAS 5.0 and normalized using the quantile normalization procedure implemented in robust multiarray analysis (RMA) (Boistad *et al*, 2003; Irizarry *et al*, 2003).

### Hierarchical clustering and statistical testing of clusters

For hierarchical cluster analysis, 1239 genes with a variation across all 118 samples greater than 0.5 were median-centred to a magnitude of 1. Samples and genes were then clustered using average linkage clustering with a modified Person correlation as similarity metric. The cluster dendrogram was visualised with Tree View (Eisen *et al*, 1998).

Clusters formed based on correlations do not provide any information concerning statistical significance. The significance of the tumour clusters generated and of each gene separately was performed as described in Supplementary data 1.

### Microsatellite status classifier

We build a maximum likelihood MSI classifier with a ‘leave-one-out’ cross-validation scheme basically as described (Dyrskjot *et al*, 2003). Only those 5082 genes with a variance across all 118 samples above 0.2 were included. We used a normal distribution with the mean dependent on the gene and the group. For each gene, we calculated the variation between the groups and the variation within the groups to select genes with a high ratio between these. To classify a sample, we calculated the sum over the genes of the squared distance from the sample value to the group mean, standardised by the variance, and assigned the sample to the nearest group. The sample to be classified was excluded when calculating group means and variances. For the final classifier we selected genes that were among those that performed best in the cross-validation test, and that represented both up- and downregulated genes.

#### Quantitative PCR

The web-based assay design software from (Exiqon™) (www.probelibrary.com) was used to design intron-spanning primer pairs and to select appropriate hybridisation probes from the Human Probe Library (Exiqon™). The hybridisation probes of the Probe Library uses a unique nucleotide chemistry called locked nucleic acid (LNA). In practice the LNA probes function as classical TaqMan probes, but because of the LNA properties they are much shorter, only 8–9 bases. A description of primers and probes can be found in Supplementary data 2. The PCR procedures were performed as described previously (Birkenkamp-Demtroder *et al*, 2002). All samples were normalised to GAPDH as this gene shows minimal expression variation in colorectal cancer samples (Andersen *et al*, 2004).

### Classification of new independent test samples based on real-time PCR

For this test, we used an independent test set comprising 47 stage II tumours of unknown MS status at the time of testing. The microarray-defined signature was translated to a PCR platform by analysing the nine-gene signature by quantitative PCR on a subset of 18 of the 101 tumour samples. The average for each gene and group of the microarray data was multiplied with a constant so that the total average was equal to the average of the corresponding log2 transformed PCR values. This translation can be made because the normalised PCR values are expected to be proportional to the normalised array values, and on a log scale this becomes an additive difference. The difference is gene specific and is therefore estimated for each gene separately. Thus, the variation obtained from the microarray data, and used for classification, can be used directly on the PCR platform.

## RESULTS

### Hierarchical clustering

**Figure 1:**
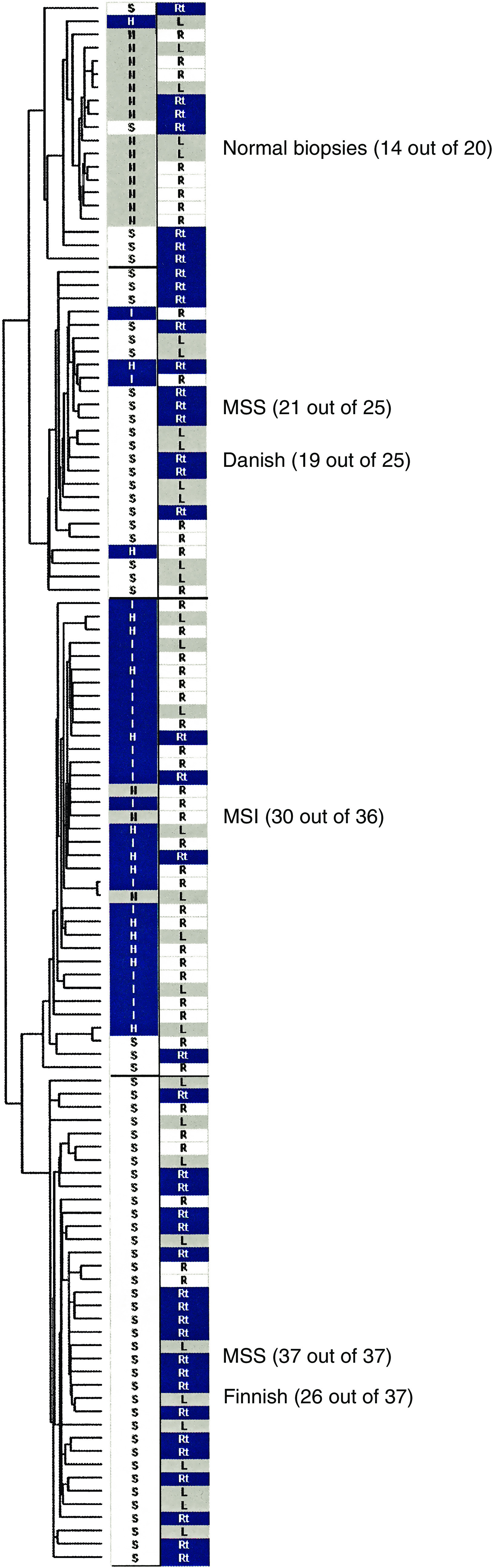
Unsupervised hierarchical clustering of 101 colorectal tumours. The phylogenetic tree shows the spontaneous clustering of 101 tumour and 17 normal biopsies into four clusters mainly consisting of normal biopsies, MSI or MSS tumours, respectively. In the left column, the microsatellite status is indicated as MSS (S) or MSI (I). Hereditary nonpolyposis colorectal cancer tumours are indicated by (H) and normal biopsies by (N). In the second column the tumour location is indicated as right-sided (R) or left-sided (L) colon, or rectum (Rt).

We examined the gene expression profiles of 101 primary colorectal carcinomas (67 MSS, 34 MSI) and 17 normal biopsies using high-density oligonucleotide arrays representing ≈15 000 genes. Redundant probesets and probesets with a variation across all samples smaller than 0.5 were removed, resulting in 1239 genes for further analysis. The clustering algorithm essentially separated the samples into three tumour clusters and a cluster with normal biopsies (Figure 1). Two of the tumour clusters contained mainly MSS tumours (37 out of 37 and 21 out of 25) and one cluster was dominated by MSI tumours (30 out of 36). In the MSI cluster, there was no sign of separation between sporadic and HNPCC samples and right-sided and left-sided tumours were interspersed among each other.

The MSI cluster contained three morphologically normal tissue specimens and six tumours were found in the cluster dominated by normal biopsies. This may be because of an atypical tissue composition in those samples, as described previously by Alon *et al* (1999) by introducing a muscle index. We adapted this method and found that the two outlying normal biopsies had an untypical low muscle index and that the tumour sample in the tight normal cluster had an untypical high muscle index (Supplementary data 3). The five tumours flanking the normal cluster had a muscle index comparable to other tumours. None of the tumours were excluded for further analysis as variation in tumour tissue composition would allow the construction of a more robust classifier.

Another observation was that the two MSS clusters were either dominated by Danish samples (19 out of 25) or by Finnish samples (26 out of 37), indicating a systematic difference between the two countries. Based on these observations, we performed a series of statistical tests that showed that the observed separation of tumours into MSS and MSI groups, as well as into Danish and Finnish groups, was highly significant even at highly strict criteria (Supplementary Table 1).

### Construction of a signature for microsatellite status

**Figure 2:**
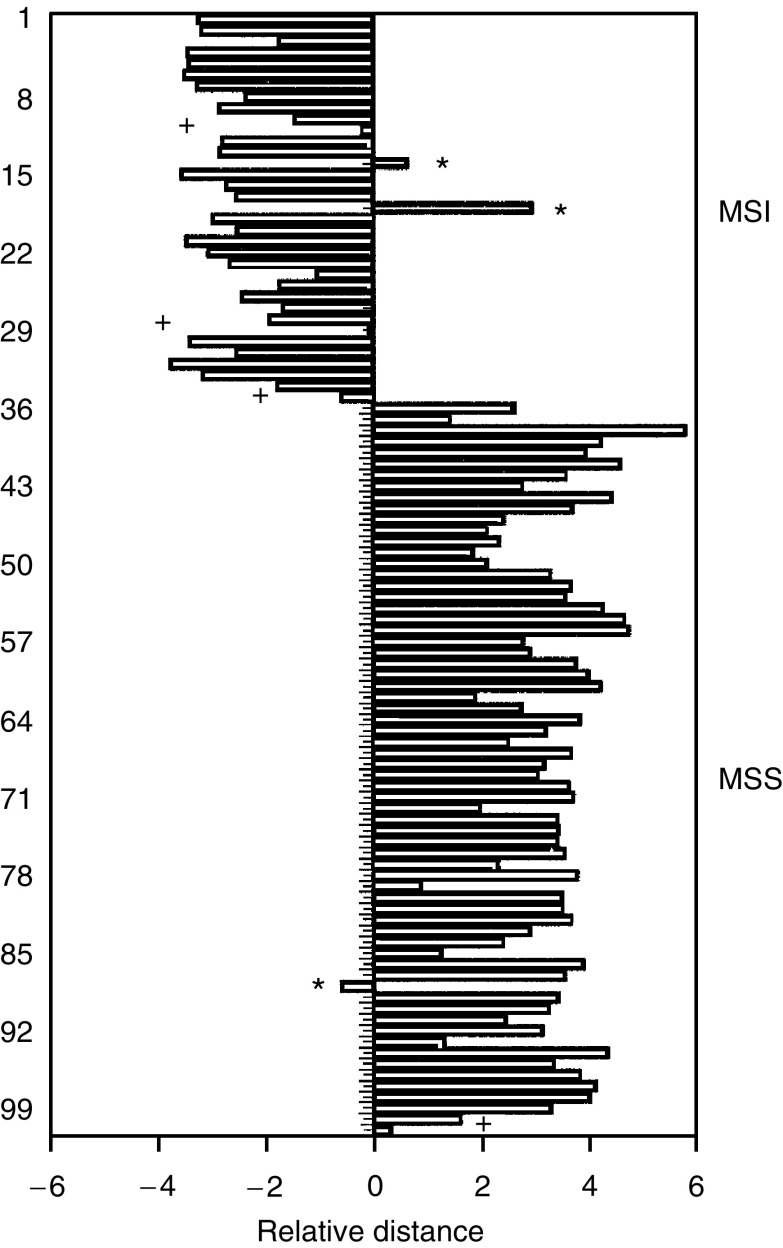
Performance of the MI classifier in the training set. The bars indicate the relative distance of every single tumour to the centres of the microsatellite unstable and microsatellite stable groups. The distances are log_2_ and defined through the cross-validation steps. A value of +2 indicates that the distance of a tumour to the microsatellite unstable group is four times the distance to the microsatellite stable group. The upper 34 tumours (open bars) are MSI tumours and the solid bars are MSS tumours. (^*^) Indicate samples that are always misclassified, and (+) indicate samples that are almost equally close to both groups.

To define a signature for microsatellite status, we used state-of-the-art supervised classification methods. We built a maximum-likelihood classifier using the 101 tumour samples and evaluated the classifier using a ‘leave-one-out’ cross-validation scheme. For classification, we selected those predictive genes that performed best in cross-validation and showed the largest possible separation of the two groups. Each tumour was classified according to its proximity to the mean of the three groups. We tested the classifier's performance using 1–100 genes in cross-validation loops, and obtained the best correlation to microsatellite status by using a 15-gene cross-validation scheme. For the final signature, we selected nine genes that were used in at least 70% of the cross-validations, and that represented both up- and downregulated genes (Table 3). With these nine genes, a correct classification was obtained in 98 tumours (97%) out of the 101 (Figure 2).

By including samples from both Finland and Denmark, we identified genes that could discriminate between MSS and MSI, independent of the geographical origin of the two groups. The genes we used for the classifier were a subset of the highest scoring genes for MSS/MSI difference for both the Finnish and the Danish samples, and they have no separating power between Finnish and Danish samples.

### Cross-platform validation

**Figure 3:**
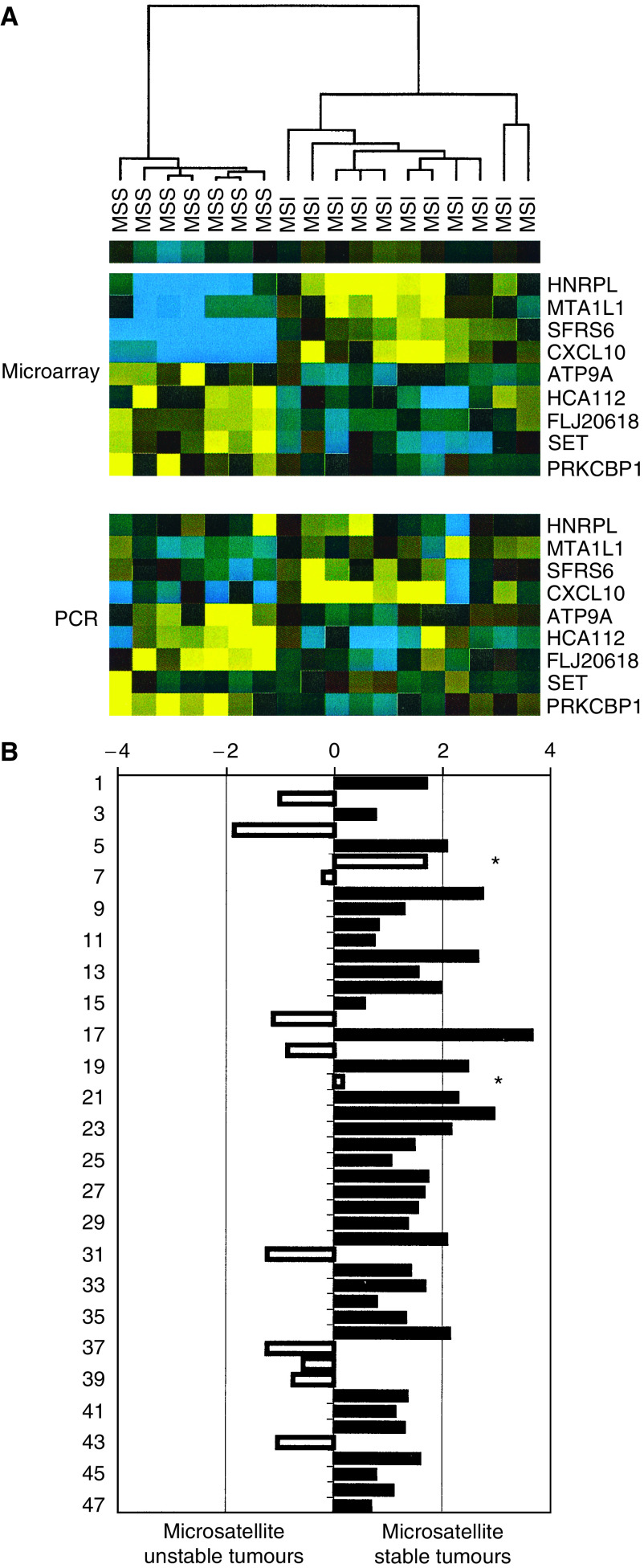
Classification of MI status based on real-time PCR. Panel A shows a cluster analysis of a subset (18 samples) of the 101 tumour samples using the nine signature genes, based on either the microarray data or the real-time PCR data. Blue colours indicate relative low expression and yellow colour high expression. Panel B shows the classification result of 47 new independent samples based on PCR data using seven of the nine genes. Relative distances are explained in the legend to Figure 2. The two misclassified tumours are indicated with an asterisk. For PCR primers and hybridisation probes, see supplementary data 2.

We next measured the gene expression level of the nine classifying genes using real-time RT-PCR. We randomly chose seven MSS and 11 MSI samples from the training set, and compared the PCR data with microarray data using clustering. Median-centred and scaled PCR data gave the same overall picture as clustered array data from the 18 samples (Figure 3A). As the genes *SET* and *ATP9a* did not work well in the PCR reaction, we used only seven of the nine classifier genes (*HNRPL*, *MTA1LI*, *SFR6*, *CXCL1O*, *HCA112*, *FLJ20618* and *PRKCBP1*) in our final RT-PCR-based classifier. We quantified the transcripts from these seven genes by real-time RT-PCR in a new independent test set consisting of 47 tumours, 35 MSS and 12 MSI tumours. Using this approach, the classification of 45 of 47 tumours was consistent with MS analysis. The two misclassified tumours were almost equally spaced between the groups of MSI and MSS tumours (marked ^*^ in Figure 3B).

### Relation between microsatellite status, stage and survival

Recent data have shown a relation between MSI classification and a good prognosis in stage II patients.

**Figure 4:**
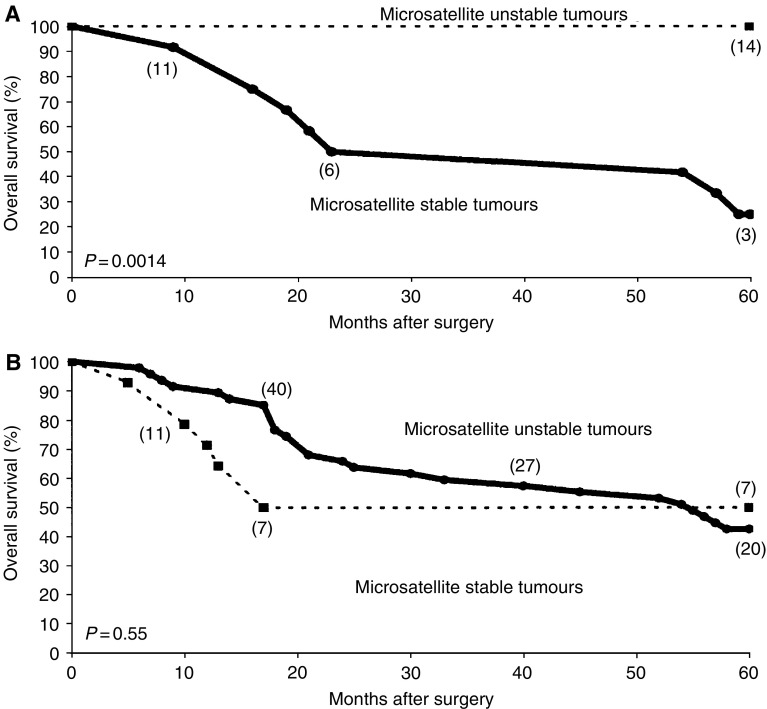
Kaplan–Meier estimates of crude survival among patient with Stage II and Stage III colorectal cancer, according to microsatellite status of the tumour determined by a nine-gene expression signature. Open triangles indicate censored samples. The patients left at risk are denoted in brackets. The *P*-values were calculated with use of the log-rank test. (**A**) Patients with Stage II colon cancer (not adjuvant chemotherapy). (**B**) Patients with Stage III colon cancer (adjuvant chemotherapy).

To examine if our tumour material was consistent with this, as well as to demonstrate a possible use of MSI classification (be it based on gene expression or microsatellites) in a clinical setting, we correlated our classification to the overall survival of the patients. We used the MSI classification data we generated on the training set with the nine-gene classifier, and Kaplan–Meier plots were constructed for stage II and III tumours separately (Figure 4). The overall survival was highly significantly related to the classification in 36 stage II patients, as 10 out of 11 patients that died within five years belonged to the MSS group (*P*=0.0014) (Figure 4A). Thus, in accordance with other recent publications, the classifier clearly proved to be a strong predictor of survival in stage II disease.

Among 65 patients with Stage III tumours receiving adjuvant chemotherapy, 16 were classified as MSI tumours and 49 as MSS tumours. As six MSI and 30 MSS patients died within five years of follow-up, there was no significant difference in overall survival between these groups (*P*=0.55) (Figure 4B).

### Construction of a classifier for sporadic MSI *vs* HNPCC

**Figure 5:**
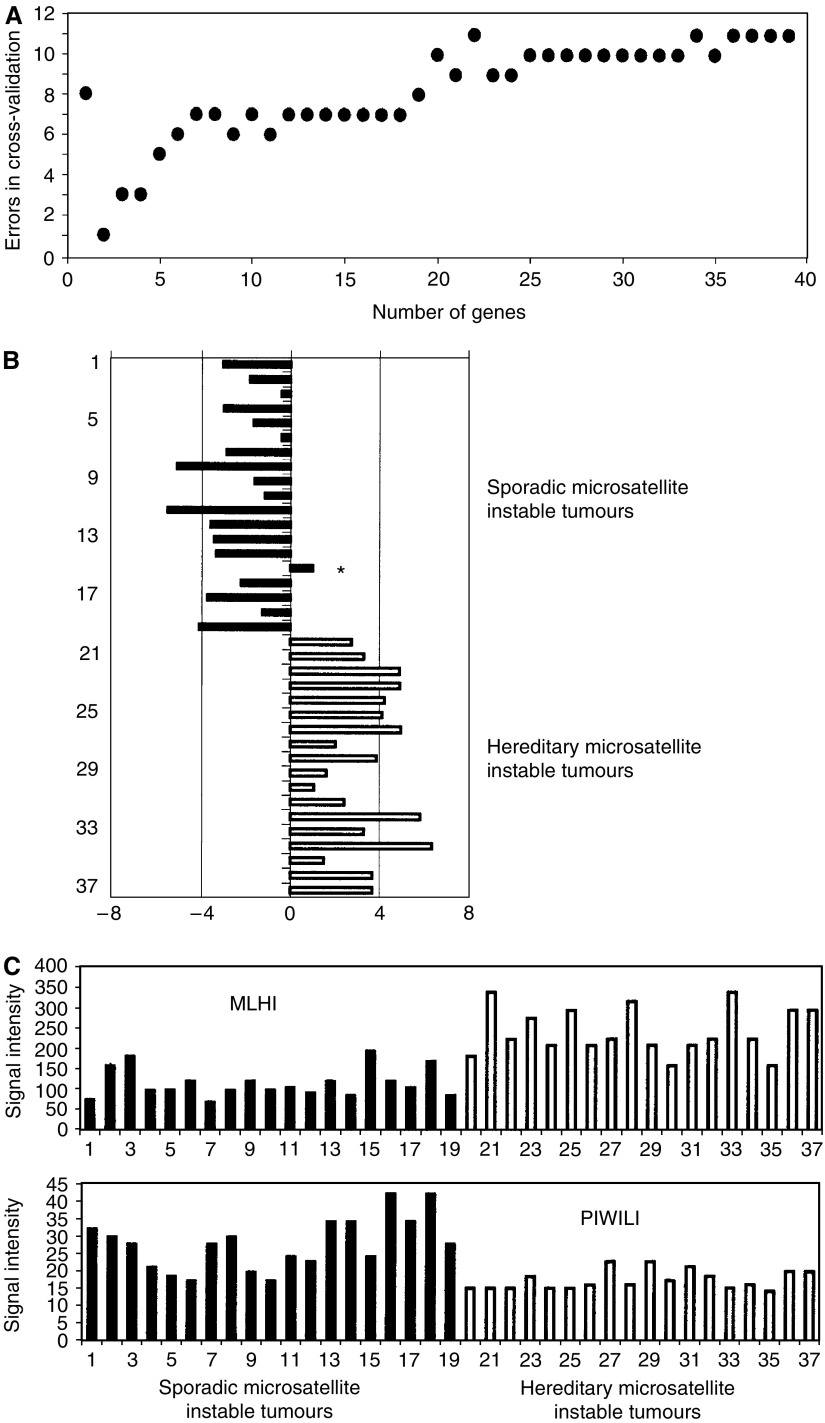
Classification of MSI tumours as hereditary or sporadic. Panel A shows the number of classification errors in cross-validation as a function of the number of genes used. The minimum number of errors found was one using two genes, and adding more genes increased the number of errors. Panel B shows log_2_ of the ratio of the distance between a tumour to the centres of the sporadic microsatellite unstable group and the hereditary microsatellite unstable group. Panel C shows microarray signal values for MLH1 and PIWIL1 genes for all tumours. Asterisk indicates the misclassified tumour.

The group of patients with MSI tumours includes both sporadic and inherited (HNPCC) cancers. As the inherited cases need an extensive clinical genetic examination of the family, a signature for this group of patients would be clinically relevant. We therefore sought for genes whose expression would identify such inherited cases. We subjected the 18 sporadic MSI samples and 16 HNPCC samples plus additional three samples (one MSI (stage I) and two HNPCC (stage I and IV)) to supervised classification as described above. The smallest number of errors in cross-validation was one error obtained when using two genes only (Figure 5A, B). In 36 of the 37 cross-validation steps, the two genes used were MLH1 and PIWIL1. These two genes are also the two genes having the largest positive (4.21) and negative (−4.89) values of the *t*-statistics for difference between the two groups. As the number of MSI tumours available was limited, we could not analyse an independent test set. We therefore made an extensive mathematical testing. To evaluate the significance of the two genes, we randomly permuted the group labels MSI and HNPCC 500 times and for each permutation calculated the 22.215 *t*-values for difference between the two pseudo groups, and recorded the maximum of the absolute values. The largest absolute value among these 500 maxima was 4.37, showing that the *t*-value -4.89 for PIWIL1 was highly significant. Only two of the following maxima were larger than 4.2, which showed that the MLH 1 *t*-test value of 4.21 had a *P*-value of less than 1%. Thus, the confidence was better than 99% in case of MLH1, and even much higher in case of PIWIL1. The mismatch repair gene *MLH1* showed a general downregulation in sporadic disease, whereas PIWIL1 was lower expressed in hereditary cases (Figure 5C).

## DISCUSSION

The main objective of this study was to build a robust gene expression classifier for MSI reflecting MMR deficiency in colorectal cancer. Our first step was to perform an unsupervised cluster analysis of tumour samples and normal biopsies. Normal samples readily separated from tumour samples, and the tumour samples spontaneously separated into MSS and MSI. This demonstrates that MSI has a profound effect on the gene expression pattern of colon cancer.

Surprisingly, the geographic origin of the patients also had an influence on the transcriptional pattern in the tumours. This was reflected in two MSS clusters, each dominated by either Danish or Finnish tumours. As the samples were labelled in mixed batches, we could exclude that the country difference was due to a labelling effect. The gene expression data in this study were derived from macroscopically removed pieces of resected tumours. The tissue used for RNA isolation was subsequently inspected by a trained pathologist and was estimated to contain from 50% tumour cells and up to more than 95%. A difference in the composition of cell types in the Danish and Finnish samples seemed unlikely, as we found no difference in muscle index (Alon *et al*, 1999). Thus, we did not find any explanation for the country effect, but speculate that it could be due to differences in tissue sampling (e.g. ischaemic time), or less likely, genetic difference or nutritional differences between Denmark and Finland. However, the main observation was that unsupervised clustering gave a clear separation between MMR-proficient and -deficient tumours.

By use of a supervised classification method, we found that using only nine genes the MSI/MSS classification was extremely robust, reaching a sensitivity and specificity that was comparable to microsatellite analysis. On a subset of the 101 tumours, we demonstrated that the signature could be transferred to a real-time RT-PCR platform. We therefore used this platform to classify an independent set consisting of 47 tumours of unknown microsatellite status. Forty-five (∼96%) were classified in consistence with subsequent microsatellite analysis; one of the misclassified tumours was clearly inconsistent, whereas the other was classified with low confidence. Tumours that are problematic to allocate to either MSS or MSI may display intratumour heterogeneity (Jimenez *et al*, 2003) or they may follow alternative genetic pathways (Smith *et al*, 2002; Tang *et al*, 2004). However, the clear separation of the tumours into the MSI/MSS classes in the vast majority of cases indicated that the assay was robust and that technical demanding and time-consuming microdissection was not necessary.

In the present study, only one case of the hereditary MSI tumours was caused by a MSH2 mutation. However, this tumour was classified correctly as microsatellite unstable. It is not known how many sporadic MSI tumours are that caused by MLH1 hypermethylation, but based on the expression levels of MLH1 and reports from the literature a realistic estimate would be 80–90% (Kane *et al*, 1997; Cunningham *et al*, 1998b; Herman *et al*, 1998; Kuismanen *et al*, 2000). The high concordance of 97% between microsatellite analysis and the gene expression classifier indicated that microsatellite unstable tumours were classified correctly, regardless of which mismatch repair genes that was inactivated.

We were able to classify MSI tumours into sporadic and hereditary cases based on the expression of only two genes, *MLH1* and *PIWIL1*. The classification resulted in only one misclassification out of 37. Analysis of this case for mutations in MLH1 and MSH2 and for the Finnish founder deletion in exon 16 of MLH 1 was negative, but were have not explored for deletions in MSH2, which is an important cause for HNPCC (Wijnen *et al*, 1998). The patient did not have any family history of colorectal cancer, but the family was very small and the patient's young age of 32 speaks for the possibility of a missed HNPCC case. A majority of sporadic and about half of hereditary microsatellite unstable colorectal cancers are caused by inactivation of MLH1 (Herman *et al*, 1998; Wheeler *et al*, 1999). In sporadic tumours this is mostly caused by biallelic promotor hypermethylation, whereas somatic mutations or loss of heterozygosity of the wild-type allele are significant mechanisms in hereditary tumours. As a result, the MLH1 expression level in sporadic cases is strongly compromised, whereas one or two alleles of MLH1 in HNPCC cases are transcribed, although encoding a mutated protein. In HNPCC cases with inactivation of *MSH2* or *MSH6* genes a correct classification would be expected because MLH1 is normally transcribed in these tumours. The second classifier gene *PIWIL1* is a member of the human Argonaute family that contain a conserved RNA-binding PAZ domain and may be involved in the development and maintenance of stem cells through the RNA-mediated gene-quelling mechanisms associated with DICER (Sasaki *et al*, 2003; Yuan *et al*, 2003). The association of this gene with hereditary MSI tumours is novel, as no biological differences between sporadic and hereditary MSI tumours have been reported. As the number of samples with MSI was limited, we only attempted mathematical validation of the two genes as predictors of hereditary MSI. It is important that other researchers repeat our finding on independent materials. If consistency is found across several materials this could be of great practical importance, as clinical genetic examination of large families is very costly. By using a classifier of heredity, efforts could be focused to those with a very high likelihood of being HNPCC cases. Recent publications indicate that classification of MMR-deficient tumours could be of use when selecting chemotherapy in stage II patients (Ribic *et al*, 2003). However, there are conflicting reports on the relation between MSI status and outcome of fluorouracil-based adjuvant chemotherapy. Some authors report that stage III MSI cancers derive the greatest benefit (Elsaleh *et al*, 2000), others that MSI status is not predicting response (de Vos tot Nederveen Cappel *et al*, 2004). These studies have recently been challenged by a large study showing that only MSS patients derived a benefit from the treatment (Brezden-Masley *et al*, 2003). In a study where 5-fluorouracil treatment failed, antitumour activity of irinotecan could be documented in 15–30% of the patients (Cunningham *et al*, 1998a; Rougier *et al*, 1998), and MSI has recently been shown to be a predictive factor for response to irinotecan in patients with advanced colorectal cancer (Fallik *et al*, 2003). The survival of the patient in this study shows the same clear trend reported by Ribic *et al* (2003). MSI in stage II patients receiving no chemotherapy is a beneficial prognostic marker, whereas MS status in stage III patients receiving chemotherapy has no prognostic values. Our gene expression signature of only nine genes thus identified a group of stage II patients that were MSI and had a very good prognosis, and a group that were MSS and had 50% mortality.

The approach described here, to detect MSI in a first step and to identify HNPCC in a second step, if confirmed in other studies, is an improvement compared to previous strategies. In the near future, molecular classification of CRC into MSI and MSS subtypes may become a routine procedure because of the emerging different treatment strategies (Gryfe *et al*, 2000; Fallik *et al*, 2003). This will introduce the problem of HNPCC detection; MSI cases detected with previous methods need careful and laborious evaluation to correctly assess the possible risk of hereditary cancer. Our approach is unique in correctly separating MSI and MSS cancers, and in addition separating hereditary MSI from sporadic cases.

The present gene expression signatures may, supplemented with future signatures, form the basis for developing a universal colon cancer diagnostic microarray capable of classifying tumour origin, type, stage and propensity to metastasise, as well as predicting their response to chemotherapy and likelihood of recurrence. We have demonstrated that such microarray-based assays may also be transferred to alternative platforms such as real-time RT-PCR.

## Figures and Tables

**Table 1:** Summary of clinicopathological and microsatellite features of colorectal cancer samples used for construction of the classifier (training set)

			**Localisation in colon**		**Tumour stage** ***N* (Danish, Finnish)**	
**Patient group**	***N* (Danish, Finnish)**	**Median age (range)**	**Right (Danish, Finnish)**	**Left (Danish, Finnish)**	**II**	**III**
All samples	118 (44,75)	62.0 (32–87)	44 (7,37)	74 (36,38)	36 (14,22)	65 (23,42)
Normal^a^	17 (6,11)	57.7 (34–80)	10 (3,7)	7 (3,4)	NA	NA
Sporadic microsatellite unstable tumours^b^	19 (4,15)	66.8 (44–87)	11 (3,8)	8 (1,7)	8 (2,6)	11 (2,9)
Hereditary microsatellite unstable tumours^b^,^c^,^d^	15 (3,12)	49.6 (*32*–*75)*	6 (1,5)	9 (2,7)	10 (2,8)	5 (1,4)
Microsatellite stable tumours^b^	67 (30,37)	61.0 (36–85)	12 (0,12)	55 (30,25)	18 (10,8)	49 (20,29)

NA=not applicable.

a Normal biopsy taken from the resection edge of a tumour.

b According to microsatellite analysis.

c According to Amsterdam Criteria 1.

d With known germline mutations.

**Table 2:** Summary of 47 Danish stage II colon cancer samples used as independent test set for the microsatellite classifier

			**Localisation in colon**	
**Patient group**	***N***	**Median age (range)**	**Right**	**Left**
All cases	47	69.0 (36–84)	11	36
Sporadic microsatellite unstable tumours^a^	37	69.0 (36–84)	7	30
Microsatellite stable tumours^a^	10	69.5 (39–81)	4	6

a According to microsatellite analysis.

**Table 3:** Genes used for the classification of microsatellite status

			**Array signal^a^**	
**Genechip probe ID**	**Gene name**	**Gene symbol**	**Microsatellite stable tumours**	**Microsatellite unstable tumours**
202072	Heterogeneous nuclear ribonucleoprotein L	HNRPL	208±73	776±340
203444	Metastasis-associated 1-like 1	MTAILI	45±13	104±36
206108	Splicing factor, arginine/serine-rich 6	SFRS6	74±56	478±242
204533	Chemokine (C-X-C motif) ligand 10	CXCLIO	111±80	315±535
212062	ATPase. class II, type 9a	ATP9a	588±222	208±114
218345	Hepatocellular carcinoma-associated antigen 112	HCAI 12	1261±603	446±271
224444	Hypothetical protein FLJ20618	FLJ20618	776±193	338±168
213047	SET translocation (myeloid leukaemia-associated)	SET	1351±298	478±201
209048	Protein kinase C-binding protein 1	PRKCBPI	294±113	158±79

a Array signal are median signal intensity values±standard deviation.
