# Supplementary material for: Gene expression signatures for colorectal cancer microsatellite status and HNPCC
Source: Br J Cancer. 2005 May 24;92(12):2240–8. doi: 10.1038/sj.bjc.6602621 (PMC2361815; doi:10.1038/sj.bjc.6602621)

Supplementary Data 1

Group testing

We make a statistical test where the p-value is evaluated through permutations. For each group and gene we calculate the average and the sum of squared deviations from the average. We then sum these over the genes and the groups:

$$S_1 = \sum_{groups} \sum_{genes} (X_{ij} - \bar{X}_{gr(i)j})^2$$

This expression is calculated for joining Danish (DK) with Finnish (SF) and MSI with MSS such that we end up with two groups. The sum of squared deviations is denoted S_2. As a test statistic we use S_1/S_2. A small value indicates that there is a real reduction in the deviations when going from 2 to 4 groups and thus the groups have a real significance. To judge if a value is significantly small we use permutations. For each of the four groups left when joining DK and SF we randomly allocate the members to a pseudo DK and pseudo SF in such a way that the number of members in each group are as in the original data

Permutation test of groups

Pseudo group	S1/S2 from data	Smaller values in 100 permutations	Minimum in 100 permutations
DK-SF	0.9146	0	0.9660
MSI-MSS	0.9084	0	0.9631

Our test value S_1/S_2 was 0.914 for Danish and Finnish samples, and 0.908 for the MSI and MSS groups, as compared to minimum values of 0.966 and 0.963, respectively, in 100 permutations. This clearly demonstrated a significant separation between the groups.

To get an understanding of this separation we performed a test to see if this is caused by few genes or if many genes are involved. For this test we calculated $S_1 = \Sigma_{genes} S_1(gene)$ and similarly with $S_2 = \Sigma_{genes} S_2(gene)$. For each gene j we used the test statistic $S_1(j)/S_2(j)$.

Permutation test of genes

		$S_1(j)/S_2(j)$			
Pseudo group		< 0.6	< 0.7	< 0.8	< 0.9
DK-SF	number of genes	22	114	425	1536
	max in 100 permutations	0	1	4	132
MSI-MSS	number of genes	49	151	461	1600
	max in 100 permutations	0	0	13	251

The clear distinction between the groups was caused by many genes.

Supplementary data 2

Target: name	Probe: name	Probe: sequence	Probe: length	Primer: name	Primer: sequence	Primer: length	Amplicon size	Intron spanning
ATP9A, ATPase, Class II, type 9A	[illegible]	[illegible]	[illegible]	ATP9A forward	agacctggcactggctcat	19	99	YES
				ATP9A reverse	[illegible]	[illegible]		
SET, translocation (myeloid leukemia-associated)	[illegible]	[illegible]	[illegible]	SET forward	agaaaaacctgctcccttcg	20	61	NO
				SET reverse	[illegible]	[illegible]		
CXCL10, chemokine (C-X-C motif) ligand 10	[illegible]	[illegible]	[illegible]	CXCL10 forward	gacattcctcaattgcttagaca	23	134	YES
				[illegible]	[illegible]	[illegible]		
HCA112, hepatocellular carcinoma-associated antigen	[illegible]	[illegible]	[illegible]	HCA112 forward	atttcgcagcttctggcatct	20	79	YES
				[illegible]	[illegible]	[illegible]		
FLJ20618,hypothetical protein	[illegible]	[illegible]	[illegible]	FLJ20618 new forward	gacaabgactgagcaagcac	20	117	YES
				[illegible]	[illegible]	[illegible]		
MTA1L1, metastasis-associated 1-like 1	[illegible]	[illegible]	[illegible]	MTA1L1 forward	caatcctgtggtgtttgtgg	20	69	YES
				[illegible]	[illegible]	[illegible]		
SFRS6, splicing factor, arginine/serine-rich 6	[illegible]	[illegible]	[illegible]	SFRS6 forward	tggaagcagatcbaggtctc	20	100	YES
				[illegible]	[illegible]	[illegible]		
HNRPL, heterogeneous nuclear ribonucleoprotein L	[illegible]	[illegible]	[illegible]	HNRPL forward	attctcaggcaaaagtgagc	20	100	YES
				[illegible]	[illegible]	[illegible]		
PRKCBP1, protein kinase C binding protein 1	[illegible]	[illegible]	[illegible]	PRKCBP1 forward	atgaagtcctgcacccagtc	20	64	YES
				PRKCBP1 reverse	[illegible]	[illegible]		

Supplementary Data 3

AFFY_ID	*GENENAME*	Mean Normal	Mean Tumor	Fold Change	T-test
201497_x_at	myosin, heavy polypeptide 11	4881	672	-7.3	1.3E-14
202274_at	actin, gamma 2, smooth muscle, enteric	1990	242	-8.2	1.7E-13
203766_s_at	leiomodin 1 (smooth muscle)	562	107	-5.2	6.7E-13
203951_at	calponin 1, basic, smooth muscle	1111	133	-8.4	1.1E-14
204083_s_at	tropomyosin 2 (beta)	770	179	-4.3	3.2E-10
204288_s_at	Arg/Abl-interacting protein ArgBP2	343	156	-2.2	1.0E-06
204570_at	cytochrome c oxidase subunit VIIa polypeptide 1	251	120	-2.1	1.8E-08
205577_at	phosphorylase, glycogen; muscle	80	54	-1.5	1.2E-09
205610_at	myomesin 1 (skelemin) 185kDa	83	55	-1.5	6.9E-10
206116_s_at	tropomyosin 1 (alpha)	1049	349	-3.0	5.6E-09
207317_s_at	calsequestrin 2 (cardiac muscle)	191	89	-2.1	6.8E-13
207390_s_at	smoothelin	792	259	-3.1	1.7E-10
212082_s_at	myosin, light polypeptide 6	3808	2125	-1.8	1.4E-13
212730_at	desmuslin	948	126	-7.5	8.0E-16
219728_at	titin immunoglobulin domain protein (myotilin)	26	20	-1.3	5.7E-10
219772_s_at	small muscle protein, X-linked	76	40	-1.9	7.6E-12

Table 1.

Based on the Go Annotation Database, we identified 16 smooth muscle related genes with high difference between tumours and normal tissues (t-test) (table 1). As described by Alon et. al, (Proc Natl Acad Sci U S A, 96, 6745-50, 1999), we calculated an average over the intensity of each gene and plotted the averaged next to the cluster dendogram (Fig 1).

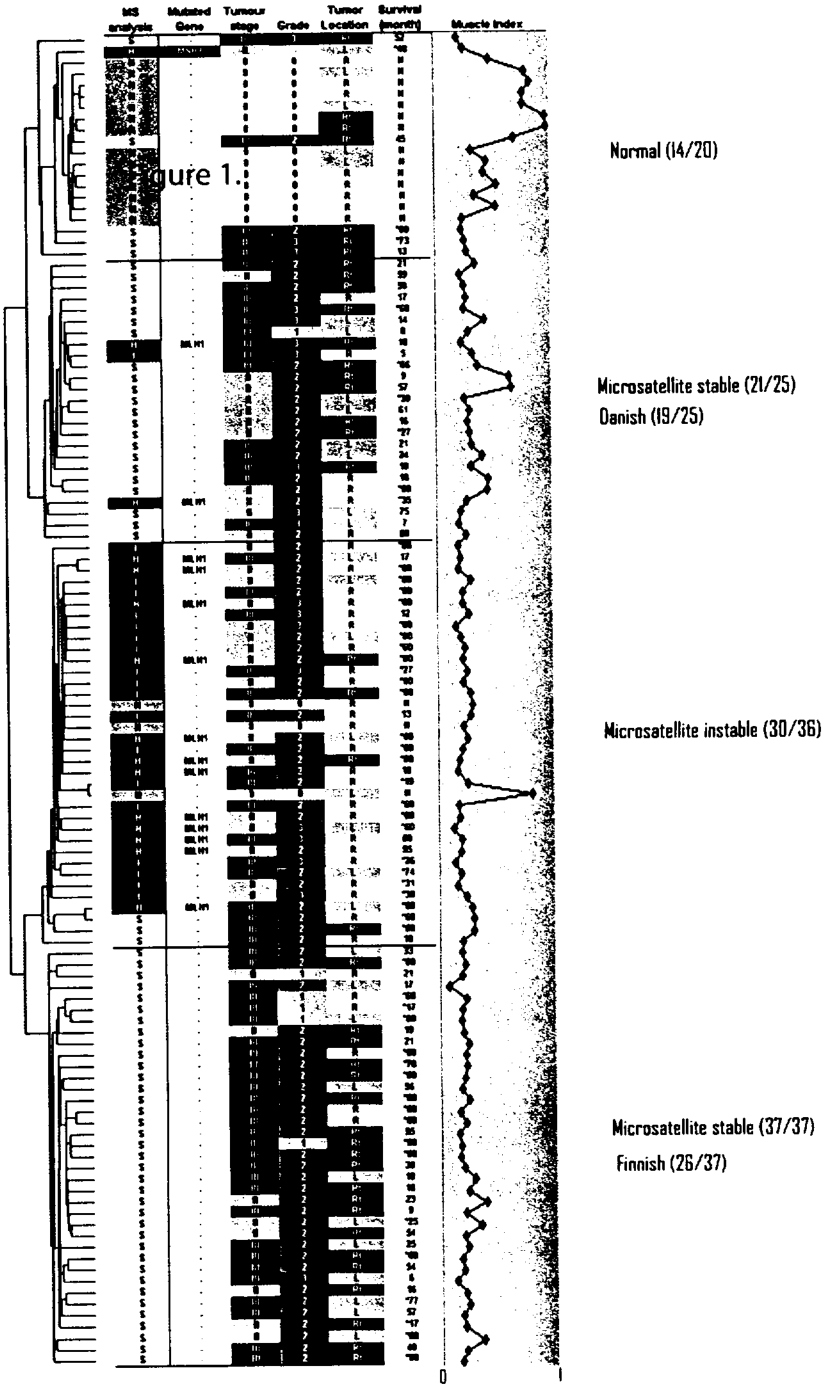

Supplement: Supplementary data [file 92-6602621x1.pdf]
